# The Immune Response to the fVIII Gene Therapy in Preclinical Models

**DOI:** 10.3389/fimmu.2020.00494

**Published:** 2020-04-15

**Authors:** Seema R. Patel, Taran S. Lundgren, H. Trent Spencer, Christopher B. Doering

**Affiliations:** ^1^Hemostasis and Thrombosis Program, Department of Pediatrics, Aflac Cancer and Blood Disorders Center, Children's Healthcare of Atlanta and Emory University, Atlanta, GA, United States; ^2^Cell and Gene Therapy Program, Department of Pediatrics, Aflac Cancer and Blood Disorders Center, Children's Healthcare of Atlanta and Emory University, Atlanta, GA, United States; ^3^Graduate Program in Molecular and Systems Pharmacology, Laney Graduate School, Emory University, Atlanta, GA, United States

**Keywords:** gene therapy, hemophilia A, inhibitors, lentiviral (LV) vector, adeno-associated viral vectors, hematopoietic (stem) cells, factor VIII (fVIII)

## Abstract

Neutralizing antibodies to factor VIII (fVIII), referred to as “inhibitors,” remain the most challenging complication post-fVIII replacement therapy. Preclinical development of novel fVIII products involves studies incorporating hemophilia A (HA) and wild-type animal models. Though immunogenicity is a critical aspect of preclinical pharmacology studies, gene therapy studies tend to focus on fVIII expression levels without major consideration for immunogenicity. Therefore, little clarity exists on whether preclinical testing can be predictive of clinical immunogenicity risk. Despite this, but perhaps due to the potential for transformative benefits, clinical gene therapy trials have progressed rapidly. In more than two decades, no inhibitors have been observed. However, all trials are conducted in previously treated patients without a history of inhibitors. The current review thus focuses on our understanding of preclinical immunogenicity for HA gene therapy candidates and the potential indication for inhibitor treatment, with a focus on product- and platform-specific determinants, including fVIII transgene sequence composition and tissue/vector biodistribution. Currently, the two leading clinical gene therapy vectors are adeno-associated viral (AAV) and lentiviral (LV) vectors. For HA applications, AAV vectors are liver-tropic and employ synthetic, high-expressing, liver-specific promoters. Factors including vector serotype and biodistribution, transcriptional regulatory elements, transgene sequence, dosing, liver immunoprivilege, and host immune status may contribute to tipping the scale between immunogenicity and tolerance. Many of these factors can also be important in delivery of LV-fVIII gene therapy, especially when delivered intravenously for liver-directed fVIII expression. However, *ex vivo* LV-fVIII targeting and transplantation of hematopoietic stem and progenitor cells (HSPC) has been demonstrated to achieve durable and curative fVIII production without inhibitor development in preclinical models. A critical variable appears to be pre-transplantation conditioning regimens that suppress and/or ablate T cells. Additionally, we and others have demonstrated the potential of LV-fVIII HSPC and liver-directed AAV-fVIII gene therapy to eradicate pre-existing inhibitors in murine and canine models of HA, respectively. Future preclinical studies will be essential to elucidate immune mechanism(s) at play in the context of gene therapy for HA, as well as strategies for preventing adverse immune responses and promoting immune tolerance even in the setting of pre-existing inhibitors.

## Introduction

Hemophilia A is the most common severe congenital bleeding disorder. The global incidence of hemophilia A is one in 4,000 male births. The disease results from genetic defects on the X chromosome at position Xq28 that cause qualitative or quantitative deficiency of blood coagulation fVIII. Clinically, patients with severe hemophilia A (<1% normal fVIII activity) have recurrent spontaneous bleeds into joints and muscles and internal/external bleeding after injury. Over the course of repeated hemorrhagic episodes, permanent damage to joints and muscles occurs. If untreated, most patients with severe hemophilia A succumb to the disease by young adulthood.

Cloning of the *F8* gene and cDNA by a group at Genentech in the 1980's launched a new era in hemophilia drug development (1, 2). This was a monumental technical achievement, as it was the largest gene ever cloned at 186,000 base pairs in length, generating an mRNA of 9,048 nucleotides (nt). The protein encoded is 2,351 amino acids [2,332 amino acids in the mature form after removal of the activation peptide (ap)] and harbors a structure designated A1-A2-B-ap-A3-C1-C2, as defined by internal sequence homologies as well as an identical domain structure to the related coagulation cofactor, factor V. The A and C domains of fVIII and factor V share homology to ceruloplasmin and discoidin/milk-fat globule-binding proteins, respectively, and likely account for their respective roles in metal ion and lipid binding. The B domain does not share sequence homology with any known proteins and its function remains poorly understood, as it is not essential for procoagulant function. This latter observation led to the development of B domain deleted (BDD) recombinant fVIII products and utilization of BDD-fVIII cDNAs in gene therapy applications where reduced size is a benefit to genome packaging within the confines of a viral vector.

Understanding of the *F8* sequence enabled commercial development of multiple recombinant fVIII products that have been licensed for the control and prevention of bleeding in hemophilia A through fVIII infusion therapy. Although only in existence for a few decades, this mode of therapy appears to transform severe hemophilia A from a uniformly lethal disease into a manageable state with a normal life expectancy. However, in 25–35% of these hemophilia A patients (<1% normal fVIII activity), an alloantibody response develops and blocks the effectiveness of fVIII replacement therapy due to the presence of neutralizing antibodies termed “inhibitors” (3). The strongest genetic predictor of fVIII immunogenicity is the causal hemophilia A mutation itself within the *F8* locus. Mutations that result in very little to no fVIII antigen produced with <1% normal fVIII activity levels (e.g., intron 22 and 1 inversions or other null mutations) are more likely to associate with inhibitor development than missense mutations that result in cross reactive material (CRM)+ status. Other than the complete absence of protein biosynthesis via a null mutation, no other dominant genetic factors of fVIII inhibitor development have been identified.

Currently in the US, as well as other economically-advantaged countries, persons with inhibitors are treated for acute bleeding with “bypassing” agents such as recombinant activated factor VII (rfVIIa; NovoSeven, Novo Nordisk), a bispecific monoclonal antibody-based fVIII mimetic (Hemlibra, Roche) or activated prothrombin complex concentrate in both acute and prophylactic settings. A second therapeutic modality, with the goal of inhibitor eradication, is immune tolerance induction (ITI). This involves repeated administration of fVIII at schedules ranging from every day to every 3rd day and dosages ranging from 40 to 300 IU/kg. ITI is the only proven therapy for achieving fVIII inhibitor eradication and subsequent fVIII product tolerance. ITI was initially described in 1977 by Brackmann and Gormsen as the “Bonn Protocol,” which consisted of a high-dose regimen designed to induce lifelong immune tolerance toward fVIII (4). Current protocols have ITI success rates of 60–80% with prognosis correlated to pre-ITI anti-fVIII titers. However, ITI treatment comes at a high financial cost and compliance burden to the patient. As gene therapy is expected to produce a continuous supply of fVIII to the bloodstream, it seems logical to expect that gene therapy could function as an ITI-type therapy. However, the mechanism of action of ITI is not understood, and the protocols used remain off-label and experimental in nature. Therefore, the study of gene therapy-based inhibitor eradication in preclinical models is warranted.

A collection of gene therapy product candidates for the treatment of hemophilia A are rapidly progressing through clinical development. The subject population has initially been limited to adult previously treated patients (PTPs) without a history of fVIII inhibitors, the rationale being that the risk of inhibitor development is lowest in this population and no inhibitors have been observed in gene therapy clinical trials to date. However, if gene therapy continues to be restricted to this subset of hemophilia A patients, its global impact will remain limited. Although it is critical to determine the immunological risk and/or benefit of gene therapy, especially inhibitor risk, in previously untreated patients (PUPs) such as children, some of the most promising gene therapy technologies may not benefit these patients. For example, as described below, adeno-associated viral (AAV) vector-based approaches do not appear suitable for adolescents with growing livers. Importantly, as previously mentioned, gene therapy may offer the potential for inhibitor eradication, thereby providing an alternative to standard ITI. In order to accomplish these objectives, preclinical investigation into the mechanisms of the immune response to fVIII in a gene therapy setting, especially those employing novel bioengineered fVIII transgenes, is needed. Likewise, it is possible that application of gene therapies toward the fVIII inhibitor problem may require new technologies and/or approaches.

In addition to exclusion criteria, other relevant pharmacological concerns of gene therapy for hemophilia A remain. For example, there is longstanding *in vitro* and *in vivo* evidence that high-level heterologous expression of human fVIII induces the unfolded protein response (UPR), a highly coordinated and regulated mechanism designed to regulate the accumulation of “unfolded” proteins in the endoplasmic reticulum (5–13). It is important to note that the discovery of UPR was in large part a direct result of the commercial development of recombinant fVIII products. Since the original discovery, a significant amount of basic research and commercial development effort has been undertaken with the goal of avoiding or controlling UPR in the context of heterologous fVIII expression. For example, our group discovered that recombinant porcine fVIII is expressed at significantly higher levels than recombinant human fVIII due to apparent avoidance of UPR and more efficient secretion from the cell (10, 14, 15). Furthermore, this high expression property translates to greater potency in gene therapy applications (16–24). One can speculate that reduced engagement of UPR also provides a safety benefit to gene therapy applications, wherein liver toxicities (e.g., elevated liver enzyme levels) of unknown origin are being observed in clinical AAV-fVIII gene therapy trials incorporating BDD human fVIII transgenes, extremely high vector doses, and potent, liver-directed promoters. However, preclinical studies have failed to recapitulate the liver pathology, leaving this mystery unresolved (25). In addition, as the UPR can engage several inflammatory cascades (e.g., NFκB pathway), and can thereby activate innate immune responses that possess the potential to skew the liver microenvironment from tolerogenic to inflammatory, avoidance of UPR activation may be the key to inducing tolerance to transgene-produced fVIII. Indeed, there is little to no evidence illustrating a correlation between UPR activation and the onset of an immune response to transgene fVIII (11, 12). However, as these murine studies utilized human or canine fVIII, it is conceivable that species differences may have affected interactions with the UPR, thereby making it difficult to tease out a potential relationship between UPR and immune responsiveness to fVIII following AAV-fVIII gene therapy. Moreover, studies have demonstrated that AAV serotypes can differentially engage UPR (26). Nevertheless, generating an AAV-fVIII gene therapy candidate that reduces the likelihood of engaging UPR may provide both safety and therapeutic benefits for patients with hemophilia A.

Within the collection of promising gene therapy product candidates, two dominant classes are apparent. The first involves *in vivo* infusion of adeno-associated viral (AAV) vectors selected for hepatocyte tropism and engineered for hepatocyte-restricted gene expression. The second involves *ex vivo* genetic modification of autologous CD34^+^ hematopoietic stem and progenitor cells (HSPC) using lentiviral vectors (LV) engineered for hematopoietic lineage-restricted expression of a fVIII transgene, followed by administration of the manipulated autologous cell product into the patient. While both classes have demonstrated evidence of safety and efficacy in small and large animal models as reviewed herein, limited data exist addressing critical parameters relating to fVIII immunobiology.

Various animal models of hemophilia have been utilized for preclinical testing of novel drug candidates. The most common species employed are mice, rats, dogs, and sheep [for review, see (27–29)]. While naturally occurring mutations have been identified in dogs and sheep, strains of mice and rats have been genetically engineered to harbor hemophilia A-causing mutations. In addition to hemophilia A animal models, wild-type non-human primates (NHP) have been utilized in preclinical testing of recombinant fVIII product candidates as well as AAV-fVIII gene therapies. Due to the immunogenicity of fVIII product candidates in humans as well as animal models, immunocompromised animals such as NOD.Cg-Prkdc^scid^ Il2rg^tm1Wjl^/SzJ mice, referred to as “NSG” mice, also are frequently employed. Clearly, under the latter setting, no immunogenicity data are obtained. But frequently, and somewhat perplexingly from an immunogenicity perspective, these often are the penultimate preclinical studies supporting human clinical testing. The goal of this review is to present an overview of the use of animal models for predictive immunogenicity and inhibitor eradication preclinical testing of gene therapy product candidates.

### History of Gene Therapy for Hemophilia A

Recombinant viral vector technology emerged shortly after the cloning of *F8*, and the first demonstration of retroviral transfer of a human fVIII transgene into cultured cells was completed by 1990 (30). This discovery sparked preclinical investigations into the use of retroviral, adenoviral, adeno-associated viral, and non-viral gene transfer methods for hemophilia A gene therapy. Overall, gene therapy approaches can be broken into two categories in terms of the route of gene transfer. *In vivo* approaches involve the direct infusion of fVIII transgene-containing vectors into subjects. In this scenario, the vectors are expected to find target cells based on their respective tropism, transfer the genetic material into the target cells, and direct expression of fVIII for secretion into the bloodstream. Early studies supported the concept that any cell type with access to the bloodstream is capable of fVIII biosynthesis. Both AAV and LV vectors are being explored as *in vivo* approaches for fVIII gene transfer. However, in addition to similar immunological challenges that could impact AAV-fVIII gene therapy efficacy, *in vivo* delivery of LV vectors can result in high transduction efficiency of antigen-presenting cells that can reduce transduction of target hepatocytes at given doses and consequently result in downstream activation of innate and adaptive anti-viral immune responses (31). As a result, *in vivo* delivery of LV vectors is still in the primitive phase of development, with a focus on tactics to overcome this additional immunological challenge (32–35). Thus, AAV vectors are currently the leading vector in this category of gene therapy and are rapidly progressing through clinical trials (36). The second category of gene therapy approaches involves *ex vivo* gene transfer wherein cells are genetically modified outside the body prior to infusion into the subject. This approach affords greater control over the gene transfer process and validation prior to administration. Several target cell types including adipocytes, mesenchymal stem/progenitor cells, and HSPCs are being pursued for *ex vivo* gene therapy (37–40). However, infusion of transduced mesenchymal stem/progenitors and adipocytes has been less successful in preclinical studies than HSPC LV-fVIII gene therapy, and thus HSPC appears to be the leading candidate (24).

By the late 1990's, amid much public excitement, several clinical trials of gene therapy for hemophilia had been initiated using both the *in vivo* and *ex vivo* approach. Unfortunately, the results were not encouraging enough to continue clinical development of these product candidates. Direct administration of recombinant retroviral vectors failed to produce a lasting therapeutic effect (41). Moreover, treatment with an adenoviral vector resulted in fVIII expression levels >1% of the normal, though adverse events were reported. In addition, administration of autologous fibroblasts electroporated with a BDD-fVIII encoding plasmid (42) only generated plasma fVIII levels near baseline (1% of normal). One aspect of these trials that may be underappreciated is the demonstration of 0% inhibitor development following gene therapy. However, all subjects were selected for a history of treatment with fVIII-containing products and no evidence of prior inhibitor development. Thus, there likely was a strong bias against inhibitor development as these subjects were assumed to have established immune tolerance or at least non-responsiveness to human fVIII as evidenced by their clinical treatment response history. However, these assumptions were not supported by preclinical animal testing, as no established animal models of immune tolerance (or non-responsiveness) to infused human fVIII products have been described nor utilized in preclinical testing. Although ~30% of humans treated with recombinant fVIII develop inhibitors, there is not an appropriate animal model that mimics these results, as nearly all animals administered human fVIII develop inhibitors.

Overall, the failure to observe safe and durable signs of efficacy in the initial hemophilia A gene therapy clinical trials, as well as other perceived failures in the field of clinical gene therapy (e.g., insertional mutagenesis and liver toxicity), resulted in a shift from commercial development of gene therapy for hemophilia A back to the academic laboratory research setting. During this time, many advances were made in the areas of gene transfer efficiency and safety, and clinical development of gene therapies for hemophilia A resumed more than a decade later with AAV-fVIII vectors taking the lead into clinical trials [for review, see (43)].

## *In vivo* Gene Therapy for Hemophilia A

### Liver-Directed AAV-fVIII Gene Therapy

Recombinant AAV vectors are the most common gene therapy vector under clinical development for hemophilia A. The basis for their extensive utilization stems from several pharmacological properties including (i) ease of delivery through peripheral vein infusion, (ii) perceived and established safety, and (iii) selective tissue tropism. Wild-type AAV is a small, non-pathogenic, non-enveloped, helper-dependent virus of the family *Parvoviridae*. AAV genetic material primarily exists in an episomal (i.e., outside of the chromosomes) form, although native AAV is known to integrate into a genetic locus termed the AAV integration site 1 (AAVS1) (44). Recombinant AAV vectors are not believed to possess this site-specific integration property and either exist episomally or integrate at low level into a broader distribution of loci. For gene therapy applications, ~90% of the single-stranded DNA genome, excluding the two inverted terminal repeats (ITRs), is replaced with a transgene cassette encompassing a transcriptional promoter, a therapeutic transgene, and a polyadenylation signal. Due to physical size constraints of the AAV capsid, this cassette must be limited to ~4.5–4.7 kilobases (kb) to ensure complete genome packaging (45).

Human and NHP are the native host, with most individuals being infected during adolescence. While wild-type AAV is non-pathogenic, over 90% of humans are environmentally exposed to AAV and can develop adaptive immunity to AAV capsid antigens. Neutralizing antibodies (NABs) to a given AAV capsid can significantly preclude the ability of AAV vectors to reach and/or transduce target cells depending on the route of administration. Moreover, memory cytolytic CD8^+^ T cell (CTL) immunity to AAV capsid antigens can cause destruction of transduced cells, decreasing transgene expression. In addition to pre-existing immunity, AAV gene therapy can itself stimulate a naïve adaptive immune response that can subsequently prevent effective repeat dosing and long-term therapeutic benefits. Indeed, an early phase one study administering up to 1.8 × 10^12^ vg/kg AAV2-factor IX (fIX) into the skeletal muscle of hemophilia B patients demonstrated fIX expression levels above baseline in four out of eight participants despite the presence of pre-existing high titer NABs to AAV2 (46). However, in a subsequent phase 1/2 dose escalation trial, hemophilia B patients administered a high dose of liver-directed AAV2-fIX (2 × 10^12^ vg/kg) developed transient therapeutic fIX levels that correlated with adaptive immunity to AAV as well as an elevation in liver transaminases (ALT, AST) that declined following loss of fIX expression (47). Similarly, patients treated with a high dose of AAV8-fIX (2 × 10^12^ vg/kg) demonstrated transient fIX expression levels associated with an elevation in liver transaminases and an increase in AAV8 capsid-specific CD8^+^ T cells (48). All participants in this study demonstrated a similar humoral immune response to AAV. Of note, glucocorticoid therapy discontinuation was found to coincide with normal liver transaminase levels, fIX levels above baseline, and a complete absence of a detectable AAV8 capsid-specific CD8^+^ T cell response, suggesting that the initial decrease in fIX expression levels may have been due to T cell immunity to AAV transduced cells. As a result, subsequent liver-directed clinical trials for hemophilia A and B have excluded patients with pre-existing NABs to the therapeutic AAV vector and plan to treat with steroids in the event that liver transaminase levels increase or transgene expression declines. Strategies such as engineered capsids, increasing the recombinant AAV dose, capsid shuffling, and decoy capsids are being tested in animal models to allow for AAV administration where NABs exist, whether from environmental exposure or from a desire to re-dose a gene therapy (49–53). The serotypes currently used in hemophilia A gene therapy trials are AAV 3, 5, 6, 8, and hu37, or modifications of the native serotypes. Rigorous comparative immunogenicity studies of the AAV capsids and/or their payload (i.e., fVIII) have not been reported, although their tropisms and thus biodistributions likely vary and may influence the immune response. Components of the AAV vector beyond the capsid, such as stimulatory hypomethylated CpG motifs, may also influence the immune response (45, 54, 55), although again, there exists little preclinical data and virtually no information on comparative immunogenicity of CpG containing and depleted fVIII-containing vector genomes.

**Figure 1:**
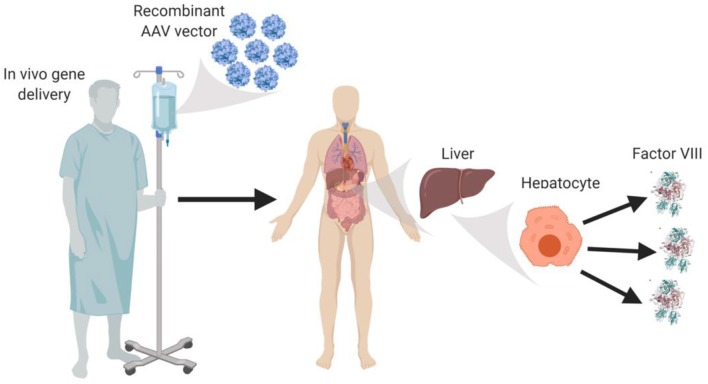
*In vivo* AAV-fVIII gene therapy. AAV-fVIII vectors selected for hepatocyte tropism and encompassing a fVIII transgene cassette under a liver-specific transcriptional promoter are infused into adult patients via peripheral vein. Once in circulation, the AAV vectors are thought to transduce primarily hepatocytes, persist episomally, and direct biosynthesis and secretion of fVIII into the bloodstream.

AAV vectors currently under clinical testing are liver-directed and employ synthetic, high-expressing, liver-specific promoters that are hypothesized to utilize the innate ability of liver protein expression to facilitate immune tolerance to fVIII. AAV-fVIII gene therapy benefits from its simplicity as it involves only a single intravenous administration of the vector (Figure 1). Thus, far, certain AAV vectors have been successful in restoring fVIII levels to the normal range and beyond, without inducing an immune response to the transgene product-derived fVIII. Despite the lack of detection of anti-fVIII antibodies, liver transaminitis occurring 6–20 weeks post-AAV-fVIII administration is a common clinical finding that appears to directly correlate with AAV vector dose (36). Although generally responsive to an extended course of high-dose steroids and transitory in nature, the molecular cause of this side effect is not understood. The medical and scientific advisory board of the National Hemophilia Foundation recently recommended that clinical trial sponsors incorporate the option of liver biopsy into clinical trial protocols to attempt to understand this phenomenon, which may have an immunological basis.

### Liver Immunobiology

With its strategically interposed organization and multicellular composition, the liver is becoming recognized and accepted as an immune organ, although it is important to point out that much of this knowledge stems from studies in animal models, mainly mice. In all mammalian species, arterial and venous blood enters the liver lobules and percolates through a honeycomb of sinusoids (capillary beds) that serve to slow the flow of blood, maximizing contact between circulating blood-borne antigens and resident immune sentinels (56). However, the liver is a unique site of blood filtration in that it must mediate clearance of potential pathogens while maintaining immune tolerance to non-pathogenic antigens. This balance between tolerance and immunity results from the complex interactions of an array of liver immune constituents, including liver sinusoidal endothelial cells (LSECs) that line the wall of the sinusoids and are intimately associated with resident macrophages of the liver (Kupffer cells), hepatic stellate cells (Ito cells) that reside in the space of Disse between hepatocytes and LSECs, and hepatic dendritic cells that reside in the sinusoidal lumen of the liver.

**Figure 2:**
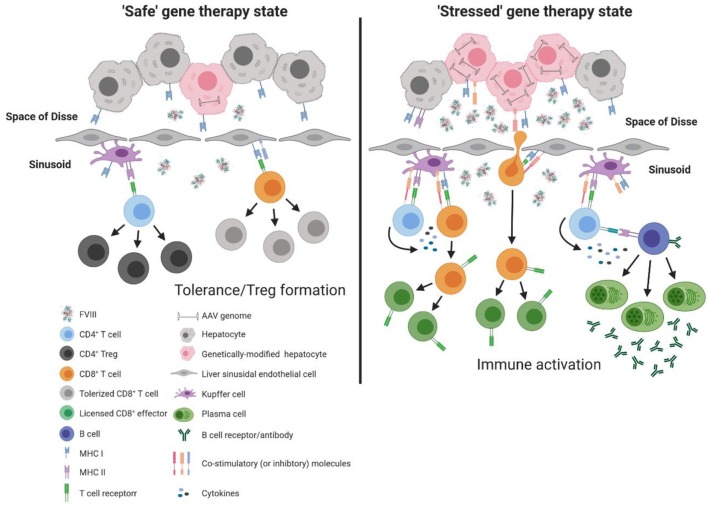
Model of immune response to liver directed AAV-fVIII gene therapy. The liver is a unique immunoprivileged site that, through complex interactions of an array of liver immune constituents, teeters between tolerance and inflammation. These immune populations include liver sinusoidal endothelial cells (LSECs) that line the wall of the sinusoids and are intimately associated with resident macrophages of the liver (Kupffer cells), hepatic stellate cells (Ito cells) that reside in the space of Disse between hepatocytes and LSECs, and hepatic dendritic cells that reside in the sinusoidal lumen of the liver. Under basal conditions, an array of immune constituents (e.g., Kupffer cells and LSECs) express low levels of MHC and co-stimulatory molecules as well as immunomodulatory cytokines. In the absence of cellular stress following AAV-fVIII gene therapy (“safe” gene therapy state), the local immunomodulatory milieu of the liver can suppress the activation of vector specific and fVIII reactive T cells. Moreover, expression of co-inhibitory molecules by LSECs can aid in the efficient differentiation of fVIII specific Tregs. However, a bolus infusion of AAV particles and/or overexpression of fVIII can lead to cellular stress that possesses the capacity to deviate the immune environment from immunomodulatory to pro-inflammatory. Under AAV-fVIII gene therapy mediated cellular stress (“stressed” gene therapy state), genetically modified hepatocytes can up-regulate MHC class I and co-stimulatory molecules as well as the production of pro-inflammatory cytokines. CD8^+^ T cell recognition of cognate antigens expressed by “stressed” hepatocytes can be activated, ultimately resulting in the cytolysis of genetically modified hepatocytes and decline in fVIII production. In addition, the pro-inflammatory milieu generated from cellular stress can promote differentiation of effector fVIII specific CD4^+^ T cells that can help activate fVIII specific B cells for formation of inhibitors.

Although each of these immune constituents are equipped with the necessary machinery to activate the adaptive immune response (e.g., major histocompatibility complex [MHC] and co-stimulatory molecules), under basal conditions these immune populations are poor activators of T cells and rather play a significant role in maintenance of T cell tolerance. This is in part due to the low expression of MHC and co-stimulatory molecules, as well as the surrounding inflammatory milieu that promotes suppression of T cell activation (57, 58). Under basal conditions, continual exposure to gut derived LPS can induce Kupffer cells to produce a variety of immunomodulatory cytokines and factors, including interleukin 10 (IL-10) and prostaglandin (PGE2) (59–61), that favor the development of regulatory T cells (Tregs) (62). Similarly, endotoxin exposure to LSECs has been shown to reduce expression of MHC and co-stimulatory molecules (63), while interaction with cognate T cells induces up-regulation of the co-inhibitory molecule, programmed death ligand 1 (PD-L1) (64). In combination with the immunomodulatory microenvironment of the liver (e.g., IL-10 and tumor growth factor β [TGF-β]), LSECs are poor activators of naïve CD4^+^ T cells but efficient at generating Tregs under basal conditions (65, 66). Moreover, LSECs have been shown to directly modulate the antigen-presentation capacity of other immune sentinels, including hepatic dendritic cells (67), that are innately “immature” due to the local milieu of the liver, and the ability of hepatic dendritic cells themselves to produce IL-10 (68, 69). These mechanisms thus collectively work to promote T cell tolerance and immune deviation from pro-inflammatory to immunomodulatory, thereby rendering the liver an attractive site for AAV-fVIII gene therapy (Figure 2). However, it is important to recognize that this balance is modulated by stimuli. Thus, we propose that a bolus infusion of ~4 × 10^15^ recombinant AAV particles (e.g., 6 × 10^13^ vg/kg dose for a 70 kg adult) predominantly transducing hepatocytes has the potential to alter the immunomodulatory status of the liver, and thereby immune responsiveness to AAV-fVIII gene therapy, through enhanced AAV exposure and/or overexpression of a protein known to induce cellular stress, such as human fVIII. For reference, the entire adult human body is thought to contain only 3.72 × 10^13^ cells (70). Therefore, in a typical expression of multiplicity of infection (MOI), this would represent a whole-body MOI of >100 and a hepatocyte-specific MOI of ~20,000!

Antigen expression by hepatocytes has been shown by multiple studies to efficiently promote antigen-specific peripheral tolerance through the development of Tregs (71, 72). There are two main categories of Tregs: naturally occurring (nTregs) and inducible (iTregs). nTregs are a distinct lineage of thymic-derived CD4^+^ T cells that account for ~5−10% of all peripheral CD4^+^ T cells. These CD4^+^ Tregs constitutively express CD25, the high affinity IL-2R (α-chain), and Foxp3 (forkhead box protein 3), a transcription factor that is crucial for the development and suppressive potential of nTregs. In mice, germline deletion of *Foxp3* can lead to a fatal lymphoproliferative disorder that can be restored upon adoptive transfer of Tregs from wild type mice (73). In addition, *scurfy* mice that possess a spontaneous recessive mutation in *Foxp3* develop a lymphoproliferative disorder that parallels IPEX (immune dysregulation, polyendocrinopathy, enteropathy, X-linked) syndrome in humans, which is also caused by mutations in *Foxp3* (74). Conversely, iTregs are generated from peripheral naïve conventional CD4^+^ T cells following recognition of cognate peptide-MHC Class II complexes in the presence of insufficient co-stimulatory signals as well as immunomodulatory cytokines (e.g., TGF-β and IL-2) and/or small molecules (e.g., retinoic acid). There are 2 predominant types of iTregs, Th3, and Tr1, both of which do not constitutively express Foxp3 nor necessitate Foxp3 for immunomodulation (75, 76). While Tr1 cells are defined by production of IL-10, Th3 cells are identified by secretion of TGF-β. The mechanisms by which Tregs can modulate immunity fall into four main categories: cell-cell contact, cytolysis, metabolic disruption, and contact independent (cytokine mediated) (77, 78). Cell-cell contact suppression operates through multiple cell surface receptors (e.g., cytotoxic T-lymphocyte associated protein 4 [CTLA-4], glucocorticoid-induced tumor necrosis factor receptor [GITR], lymphocyte activating 3 [LAG-3]) that modulate the activation of T cells and stimulatory capacity of antigen-presenting cells. In addition, Tregs can suppress immune responses through cytolytic mechanisms involving secretion of perforin and granzyme B. Metabolic disruption includes delivery of cAMP to effector T cells, as well as expression of ectoenzymes CD39 and CD73. Cytokine-mediated immunomodulation includes secretion of IL-10, IL-35, and TGF-β, and with Th3 iTregs low amounts of IL-4. Currently, the “division of labor” between nTregs and iTregs remains unclear.

Administration of hepatotropic AAV2-OVA (ovalbumin) gene therapy leads to induction and enrichment of OVA specific CD4^+^ CD25^+^ Foxp3^+^ T cells that are phenotypically and functionally characteristic of Tregs (71). Similarly, several studies demonstrate that gene transfer of human fIX using liver-tropic AAV promotes generation of Tregs that have the capacity to suppress antibody formation to human fIX following transfer into naïve hemophilia B mice (71, 79, 80). Moreover, *in vivo* removal of CD4^+^ CD25^+^ Tregs using an anti-CD25 monoclonal antibody results in the development of antibodies to human fIX following hepatic gene transfer. These results collectively support the notion that hepatic expression of antigens in mice can lead to immune tolerance through formation of Tregs. Although these mechanisms are well-described in mice, parallel mechanisms in humans remain to be defined.

### Murine Preclinical Studies

Over the past decades, several studies have demonstrated preclinical efficacy following liver-directed AAV-fVIII gene therapy (21–23, 81–87). However, these studies vary greatly in respect to pharmacological parameters including study duration, vector doses, fVIII transgene design, experimental species utilized, and/or the use of immunodeficient animals or transient immune suppression to obviate immune complications (Table 1). For example, a study by Herzog and colleagues in 2012 demonstrates that liver directed AAV gene transfer of BDD human (h)fVIII (AAV-hfVIII) can induce immune tolerance to fVIII (89). Using hemophilia A mice on a BALB/c background, data from this study demonstrate that AAV8-hAAT-hfVIII (10^11^ vg/mouse) gene therapy can not only correct fVIII levels, but also results in low to no detectable inhibitor titers following subsequent challenge with recombinant human fVIII. The ability to induce tolerance to human fVIII in these mice was found to occur in both the presence and the absence of transient immunosuppression mediated by depletion of B cells 1 week prior to AAV8-hAAT-hfVIII gene therapy. Interestingly, when the same experimental setup was replicated in hemophilia A mice on a mixed S129-C57BL/6 background, AAV8-hAAT-hfVIII gene therapy only resulted in a significantly diminished inhibitor response following subsequent challenge with recombinant human fVIII. The differential outcome observed between both strains of hemophilia A mice is similar to the disparate immune response to recombinant human fVIII that is observed in different background strains of hemophilia A mice (90). These results highlight the potential role of genetics, in particular immune polymorphisms, on whether a patient will respond to AAV-fVIII gene therapy. Further examination of the immune response to AAV8-hfVIII in BALB/c hemophilia A mice in this study demonstrated that liver-directed AAV-fVIII gene therapy in the presence or absence of B cell depletion resulted in a significant decrease in IL-2 and IL-10, and a partial reduction in IL-4 and IL-13 gene expression. Adoptive transfer of CD4^+^ CD25^+^ cells from tolerized BALB/c hemophilia A mice into BALB/c naïve hemophilia A mice was found to modestly diminish the *de novo* fVIII immune response to recombinant fVIII challenge. Given the strong evidence that the immune response to recombinant fVIII is dependent on CD4^+^ T cell help (98–100), it also is hypothesized that liver-directed AAV gene therapy may enhance production and activation of fVIII-specific Tregs that in turn can actively suppress effector T cells and B cells, allowing for sustained production and therapeutic plasma levels of fVIII.

**Table 1:** Summary of preclinical gene therapy studies for hemophilia A.

**fVIII transgene**	**Transgene species**	**Vector**	**Delivery**	**Model**	**Inhibitor status**
BDD-fVIII	Human	AAV	I.V.	Mouse	Sometimes^a^
BDD-fVIII	Human	AAV	I.V.	NHP	Yes^b^
hfVIII-N6 or -V3	Human	AAV	I.V.	NHP	Yes^c^
BDD-cfVIII	Canine	AAV	I.V.	Dog	Rarely^d^
ET3	Human/Porcine	AAV	I.V.	Mouse	Sometimes^e^
ET3	Human/Porcine	LV	HSCT	Mouse	No^f^
An53	Ancestral (95% Human)	AAV	I.V.	Mouse	No^g^
BDD-cfVIII	Canine	LV	I.V.	Mouse	Yes^h^
BDD-pfVIII	Porcine	LV	HSCT	Mouse	No^i^
BDD-fVIII	Human	LV	I.V.	Mouse	Sometimes^j^
BDD-fVIII	Human	AAV	I.V.	Dog	Yes^k^

a *Greig et al. (88); Sack et al. (89); Qadura et al. (90)*.

b *Bunting et al. (25)*.

c *Mcintosh et al. (87)*.

d *Sabatino et al. (85); Callan et al. (91); Finn et al. (92)*.

e *Lytle et al. (22); Brown et al. (21)*.

f *Doering et al. (17, 19, 24)*.

g *Brown et al. (23)*.

h *Staber et al. (93)*.

i *Gangadharan et al. (16); Ide et al. (18, 94)*.

j *Merlin et al. (95); Wang et al. (96)*.

k *Sun et al. (97)*.

It remains undetermined whether liver directed AAV gene therapy expands nTregs or shifts peripheral naïve CD4^+^ T cells toward iTreg differentiation. nTregs are thought to have poor proliferative capacity and are mostly polyclonal, with a minor population of nTregs suggested to possess a T cell receptor (TCR) specific for a single antigen (101). Moreover, only a minority of nTregs are thought to have strong suppressive activity (102). Thus, liver-directed AAV-fVIII gene therapy may predominantly be driven by an iTreg response. However, hepatotropic AAV-OVA gene transfer was found to induce OVA-specific CD4^+^ CD25^+^ Foxp3^+^ Tregs in both the periphery and thymus, suggesting that liver-directed AAV gene therapy may possess the potential to suppress inhibitor formation by promoting formation of nTregs and iTregs (71). Furthermore, unlike nTregs, iTregs are plastic and under appropriate conditions possess the ability to revert back to effectors. Characterization of whether under these conditions (e.g., pro-inflammation) tolerized AAV-fVIII treated animals can maintain non-responsiveness to fVIII necessitates investigation.

As Tregs appear to be critical to induce immune tolerance to fVIII following gene therapy, several studies have investigated different mechanisms to further expand and enhance Treg formation. One such mechanism is through the use of IL-2+IL-2R antibody complexes. IL-2 is a key cytokine that drives T cell proliferation and differentiation into effector cells. Moreover, IL-2 has been shown to be required for development of Tregs, though the exact role of IL-2 in induction of Tregs *in vivo* remains unclear (103–105). Recently, it was reported that IL-2 bound to a specific monoclonal anti-IL-2 antibody (JES6-1A12) expands CD4^+^ CD25^+^ Tregs (106) and protects against various experimental autoimmune diseases as well as rejection of an allogeneic solid organ graft (107–109). When used in conjunction with plasmid fVIII gene therapy, the IL-2+IL-2R antibody complex prevented the formation of inhibitors to fVIII and was found to associate with a five to sevenfold expansion of Tregs in secondary lymphoid organs of treated mice (110). An alternative approach that is being used to augment Treg formation following gene therapy is the co-administration of rapamycin (also known as sirolimus), a small molecule that inhibits the activity of mammalian target of rapamycin (mTOR). mTOR is a serine/threonine protein kinase that is engaged following IL-2/IL-2R ligation. Activation of mTOR promotes protein synthesis, cell cycle progression, and glycolysis. Blocking mTOR not only decreases cell cycle progression, which certainly suppresses T cell proliferation, but results in apoptosis in the presence of cognate antigen recognition. However, as Tregs express the high affinity IL-2R (CD25) and ligation of CD25 engages an alternative pathway than the mTOR cascade, rapamycin exposure actually promotes expansion of Tregs. The ability of rapamycin to selectively induce Tregs is dependent on time and dose, with studies demonstrating that alternating day treatment or withdrawal can better promote Treg proliferation compared to continuous administration. Co-administration of rapamycin with fVIII was found to inhibit T cell activation, increase CD4^+^ CD25^+^ Foxp3^+^ Treg numbers, and reduce antibody formation in naïve and sensitized mice (111). However, the utility of rapamycin in clinical AAV-fVIII gene therapy has yet to be explored.

In addition to Tregs, other mechanisms that may contribute to polarizing the immune response to immunoregulatory include programmed cell death (apoptosis), T cell anergy, and decreased antigen presentation. Hepatotropic AAV-OVA gene transfer was found to induce anergy and deletion of OVA specific CD4^+^ T cells (112). Moreover, Kupffer cells, LSECs, and dendritic cells have been shown to present liver-derived antigens following gene transfer to CD4^+^ T cells both in the liver and hepatic draining lymph nodes (72). CD4^+^ T cells and Tregs induced in the liver were then found to egress to hepatic-draining lymph nodes for further proliferation and differentiation, as indicated by *in vivo* proliferation of OVA specific CD4^+^ T cells and expansion of CD4^+^ CD25^+^ Foxp3^+^ Tregs in the draining lymph nodes. Expanded Tregs disseminate to the systemic circulation to mediate peripheral immune tolerance. Although outside the realm of AAV-fVIII gene transfer, Scott and colleagues demonstrated tolerance induction to fVIII through retroviral gene transfer of immunodominant human fVIII A2 and C2 domains fused to IgG. B cells were transduced and adoptively transferred into E16 hemophilia A mice that possess a deletion in exon 16 of *F8* (113). The transfer significantly decreased formation of inhibitors to the A2 and C2 domain of fVIII as well as T cell proliferation. Similarly, adoptive transfer of transduced B cells resulted in significant reduction in the T cell response to fVIII as well as pre-existing inhibitor titers even following additional challenges with recombinant fVIII. Depletion of Tregs using an anti-CD25 monoclonal antibody was found to significantly reduce the ability of B cell directed gene therapy to mediate immune tolerance to fVIII.

The dose of an antigen has been shown to play a role in polarization of Tregs and/or activation of an insufficient CD4^+^ T cell response that may consequently impact the ability to activate antigen experienced B cells (114–117). Low doses of high-affinity ligands in the presence of insufficient co-stimulatory signals promote iTreg generation (116). Moreover, higher expression levels of fIX in the liver have been shown to correlate with enhanced formation of Tregs and immune tolerance induction following subsequent recombinant fIX exposure in the presence of an adjuvant (79, 118). Several factors that may regulate transgene expression following AAV gene therapy include but are not limited to the vector dose, the transgene sequence, and/or the promoter/enhancer elements. Consistent with this, our group has observed that the dose of AAV-fVIII administered can influence the overall immunological outcome to fVIII exposure. Administration of a mid (4 × 10^12^ vg/kg) or high (2 × 10^13^ vg/kg) dose of our bioengineered high-expression fVIII transgene, designated ET3, driven by a liver-directed promoter (AAV8-HLP-ET3) resulted in dose dependent fVIII expression, with roughly 70% (0.7 IU/mL) and 200% (2 IU/mL) normal human fVIII levels detected, respectively (22). Both dose groups failed to generate antibodies to ET3 up to 5 months post AAV8-HLP-ET3 treatment. However, upon exposure to infused recombinant ET3, plasma fVIII activity levels quickly declined in mid-dose treated mice. The disappearance of detectable activity levels correlated with the onset of a robust anti-ET3 IgG response. Conversely, high-dose treated mice demonstrated a transient decline in plasma fVIII activity levels that increased following termination of intravenous ET3 infusion, and only one out of three recipients in this cohort harbored detectable antibodies to ET3. These data suggest that higher doses of AAV-fVIII gene therapy may facilitate immune tolerance or non-responsiveness to fVIII, but this has yet to be convincingly demonstrated and replicated. Although mice can be tolerized to fVIII through liver-directed AAV-fVIII gene therapy, wild-type NHP almost uniformly develop inhibitors to transgene-expressed human fVIII using similar technologies and approaches as those described herein. Therefore, our understanding of the tolerogenic mechanisms established through AAV-fVIII directed gene therapy remains incomplete.

The dose of AAV vector used in gene therapy is limited as there is risk of acute toxicity as well as activation of an adaptive immune response to certain vector elements including the protein capsid and transgene product. To overcome this, our group and others are investigating ways to optimize the fVIII transgene cassette to facilitate increased expression of fVIII with a lower dose of AAV vector (i.e., increased product potency). The first strategy involves the development of synthetic promoters that direct high-level expression in liver hepatocytes but no other cell types. One such promoter is designated HCB and has a minimal size of 147 bp (23). Despite the treatment of over 100 E16 hemophilia A mice on a mixed S129-C57BL/6 background with varying doses of AAV-HCB-fVIII vectors, no fVIII inhibitor development has been observed. This finding is independent of the fVIII transgene used as BDD-hfVIII, ET3, and ancestral fVIII variants (e.g., An53) all have been tested, and despite the presence of up to 10% non-human sequence, no antibody formation has been observed [unpublished data as well as (23, 86)]. In contrast, other groups clearly demonstrated inhibitor development using codon-optimized BDD-hfVIII transgenes driven by alternative synthetic promoters. For example, Wilson and colleagues compared a wide array of synthetic enhancer/promoter combinations, with some apparently demonstrating more or less inhibitor development than others (88). Collectively, these data suggest that promoter strength and/or specificity may be dominant factors in inhibitor development in the context of liver-directed AAV-fVIII gene therapy. As there are no established differences in immunogenicity among the various recombinant fVIII products, despite substantial differences in the cellular source (baby hamster kidney, Chinese hamster ovary, or human embryonic kidney cell lines), primary amino acid sequence (SNPs, ± BDD, addition of IgG Fc), and post-translational modifications (both inherent glycation as well as synthetic additions such as PEG), it seems reasonable to speculate that promoter design may be a stronger determinant of fVIII immunogenicity than transgene design and primary amino acid sequence, which should benefit clinical translation of bioengineered fVIII technologies.

Although several liver-directed AAV-fVIII gene therapies are progressing to clinical trials, most of the preclinical data supporting these trials remain unpublished. However, the team at Biomarin recently published a comprehensive preclinical dataset supporting the development of BMN 270, now referred to as Valoctocogene Roxaparvovec, an investigational AAV-fVIII gene therapy in phase three clinical trials (25). BMN 270 is an AAV serotype 5 vector encoding a codon-optimized BDD-hfVIII transgene driven by a small, liver-directed promoter termed HLP. In preclinical pharmacology studies, the authors noted “sporadic formation of anti-hfVIII antibodies was detected beyond 4 weeks post-dosing (data not shown).” Therefore, the majority of the studies performed involved the utilization of both RAG2^−/−^ mice and double RAG2^−/−^ FVIII^−/−^ mice to address issues of dose responsiveness and therapeutic efficacy. However, studies such as these do not provide any insight or prognostic value toward clinical immunogenicity of AAV-fVIII gene therapy product candidates, and brings back to light the longstanding question regarding the value and need for preclinical immunogenicity testing.

### Canine AAV-fVIII Preclinical Studies

While the genetic and immunogenic homogeneity of inbred murine models of hemophilia A allow for more precise mechanistic studies, the canine models of hemophilia A permit examination of a potentially more clinically representative immune response to liver-directed AAV-fVIII gene therapy. The canine models of hemophilia A are unique in that they can be caused by a spectrum of genetic mutations that are similar to those in patients with hemophilia A, are outbred and thereby of various genetic backgrounds, and demonstrate a bleeding phenotype that is similar to humans (119–121). Thus, canine models of hemophilia A allow for potentially more accurate examination of the therapeutic benefit of liver-directed AAV-fVIII gene therapy in humans. Currently, two primary colonies of canine hemophilia A are utilized to study the ability of liver-directed AAV-fVIII gene therapy to correct hemostasis while inducing immune tolerance to fVIII. One resides at Queen's University (QU) in Ontario, Canada and the other at University of North Carolina (UNC) at Chapel Hill. While both colonies possess a similar mutation to the human intron 22 inversion and are CRM negative (121–123), the QU colony consists of canines that are “inhibitor-prone,” with ~25% of canines developing inhibitors following exposure to canine cryoprecipitate (82). Conversely, the UNC colony appears to consist of animals that demonstrate both a low and a high propensity to develop inhibitors following canine fVIII (cfVIII) treatment; the “inhibitor-prone” canines of the UNC colony illustrate a similar frequency of inhibitor development as those from the QU colony. Similar to patients with hemophilia A, the factors that govern responsiveness in these animals remain undefined, though these differences highlight the potential contribution of genetic factors in immune responsiveness to fVIII.

Several studies demonstrate that liver-directed AAV-fVIII gene therapy can not only correct hemostasis, but also promote tolerance to fVIII. Using hemophilia A canines from the UNC colony, a study by Sabatino et al. demonstrates that one out of nine hemophilia A canines developed inhibitors following treatment with codon optimized cfVIII (85). However, the inhibitor titer was low (2.5 BU) and transient, resolving within 7 weeks of initial treatment. Subsequent challenge with recombinant cfVIII did not result in inhibitor formation, suggesting induction of immune tolerance. Interestingly, this single hemophilia A canine was later identified as a member of the newly generated “inhibitor-prone” UNC colony; introduction of an outside male breeder resulted in this subset of hemophilia A canines at UNC. These findings again suggest that AAV gene therapy has the potential to induce immune tolerance to fVIII.

### Non-human Primate (NHP) AAV-fVIII Preclinical Studies

The use of NHP provides the opportunity to examine the therapeutic efficacy of liver-directed AAV-fVIII gene therapy in a more clinically-relevant setting, particularly from the AAV tropism perspective, which is a key pharmacological parameter. However, somewhat paradoxically, unlike murine and canine pre-clinical studies at the higher end of dose range finding studies, naïve NHPs mount robust immune responses to human fVIII derived from liver-directed AAV-hfVIII gene therapy. A study by McIntosh et al. demonstrates that administration of a high dose (2 × 10^13^ vg/kg) of an rAAV8-HLP-codop-hfVIII-N6 variant (226-amino acid spacer in place of B domain of fVIII) results in peak fVIII activity levels of roughly 65% and 105% of normal human fVIII activity levels (87). Low dose (7 × 10^12^ or 2 × 10^12^ vg/kg) treatment with a disparate rAAV8-HLP-codop-hfVIII-V3 variant (replaced N6 with a 17 amino acid peptide) resulted in peak fVIII activity levels of 138% and 43% of normal human fVIII activity levels. Three out of four NHPs in this study were found to develop inhibitors (3–15 BU/mL) within 6 weeks of gene transfer. Of note, the single NHP that did not form detectable inhibitors was treated with a low dose of rAAV8-HLP-codop-hfVIII-V3. To eradicate inhibitors in these animals, the three responding NHP were treated with rituximab and cyclophosphamide. Likewise, in the BMN 270 preclinical evaluation, three out of four treated NHP mounted an anti-hfVIII immune response by 8 weeks post-AAV-fVIII administration at doses of 10^13^ vg/kg and 3.6 × 10^13^ vg/kg (25). The one NHP that did not possess measurable anti-fVIII antibodies was in the lower dose cohort.

The ability of NHP to form inhibitors following liver-directed AAV-hfVIII gene therapy is quite surprising and interesting, as the NHPs used in these studies do not have hemophilia A and endogenous NHP fVIII bears 99% sequence identity to human fVIII. Why human fVIII is immunogenic in NHP remains largely unclear. However, it is possible that the 1% difference between NHP and human fVIII generates peptide variants that, in conjunction with a different MHC (also referred to as human leukocyte antigen (HLA) in humans) profile than humans, is ultimately responsible for initiating an inhibitor response. During positive selection in the thymus, T cells that recognize self-peptide-MHC complexes with too low or high affinity are deleted, while those that have moderate affinity are provided survival signals to ensure that T cells entering the periphery have some affinity for MHC molecules (termed MHC restriction). Thus, as all peripheral T cells to some degree recognize MHC molecules, TCRs must discriminate between small differences that are provided by the cognate peptide itself, and suggests that TCRs are promiscuous. Consistent with this concept, studies using peptides with small variations (termed altered peptide ligands) demonstrate that the TCR can respond to a range of peptides that differ in fidelity to the original peptide, and that each of these altered peptide ligands can induce a spectrum of T cell responses (124, 125). While some altered peptide ligands can act as an agonist or super agonist, others can function as antagonist. Similar to the impact peptide variations can have on the overall T cell response to an immunogen, it also is possible that the MHC profile of NHP differs from humans such that it supports the appropriate presentation of human fVIII peptides to fVIII reactive T cells. Peptides utilize specific residues (amino acids) within the sequence to bind to the binding groove of MHC molecules, and it is this binding that impacts the peptide affinity to the MHC molecule. The stark contrast of the potent immunogenicity findings generated in NHP compared to non-responsiveness, at least in terms of anti-fVIII antibodies, observed in human clinical trials and mice again highlights the lack of understanding in general regarding fVIII immunogenicity and brings into question the predictive value of preclinical studies.

### Inhibitor Eradication via Liver-Directed AAV-fVIII Gene Therapy?

Recently, two independent groups demonstrated in a murine hemophilia B preclinical model that liver-directed AAV- or LV-fIX gene transfer can eradicate anti-fIX inhibitors and provide phenotypically-corrective plasma fIX activity (118, 126). One aspect of hemophilia research that sometimes appears underappreciated is the molecular or structural dissimilarity between fVIII and fIX. In this context, it should not be surprising that they possess differential risks and pathologies associated with immunogenicity [for review, see (127)]. Therefore, our group previously tested the ability of liver-directed AAV-fVIII gene therapy to eradicate fVIII inhibitors in hemophilia A mice and found that, unlike liver-directed fIX expression in hemophilia B mice, liver-directed fVIII gene therapy in the pre-immunized hemophilia A setting did not eradicate inhibitors (22). Based on these findings, we hypothesize that immune barriers in hemophilia A are greater than in hemophilia B due to differences in fVIII/fIX immunobiology. In contrast to murine studies, administration of liver-directed AAV-cfVIII resulted in undetectable inhibitor titers within 4–5 weeks post treatment in three out of three UNC hemophilia A canines with historically high pre-existing inhibitors (92). The eradication of inhibitors coincided with progressively increasing fVIII levels, improved bleeding phenotype, and improved normal pharmacokinetics to infused cfVIII. Interestingly, 1 hemophilia A canine from the QU colony had an amnestic response after gene therapy with a peak inhibitor titer of 216 BU that then became undetectable after 18 months. The immune tolerance induction in this animal was maintained even after challenge with recombinant cfVIII. Though one canine in this study did develop an amnestic response, the results from this study are promising as inhibitor titers >100 BU during ITI typically correlate with ITI failure. However, this canine tolerized rapidly compared to the years it would have taken with ITI. Immune tolerance was maintained in all canines for more than 5 years and was found to correlate with an increase in CD4^+^CD25^+^FoxP3^+^ Tregs that preceded eradication of inhibitors. Thus, while liver-directed AAV-fVIII has the potential to generate inhibitors in some canine colonies, it also can promote immune tolerance and eradicate pre-existing inhibitors in a preclinical model of hemophilia A. As the tolerance induction observed with liver-directed AAV-fVIII gene therapy in the canine model of hemophilia A utilized a cfVIII transgene, while our murine studies used human or bioengineered human fVIII transgenes, it is possible that the presentation of identical peptides as what may be recognized during central tolerance promotes induction and expansion of both iTregs and nTregs, and that liver-directed AAV-fVIII may require some aspect of a species-specific fVIII transgene.

### Caveats of Preclinical Studies

While preclinical studies of AAV-fVIII gene therapy certainly provide fundamental insight into the immune response to transgene fVIII and allow for the development of effective and safe AAV-fVIII gene therapy candidates, a major caveat that warrants discussion is the use of xenogeneic fVIII transgenes in murine and NHP preclinical studies. Unlike preclinical canine studies that utilize canine fVIII transgenes, wild type NHPs and murine models of hemophilia A are infused with AAV vectors encoding a human or bioengineered human fVIII transgene. In addition, while AAV-cfVIII gene therapy in canine models of hemophilia A results in a heterogenic immune response that more closely resembles what is observed clinically, wild type NHPs uniformly generate a robust humoral immune response to human fVIII following AAV-hfVIII gene therapy. Moreover, certain murine models of hemophilia A can develop humoral immunity to human fVIII following AAV-hfVIII gene therapy. As human fVIII shares some degree of identity with NHP and murine fVIII (99 and 87%, respectively), it is possible that unidentical peptides derived from the human fVIII in conjunction with a distinct MHC profile may contribute to whether murine and NHP models respond to transgene human fVIII. Similarly, patients with hemophilia A have distinct HLA profiles that may differentially bind to the same fVIII peptide but consequently have disparate outcomes, with the same fVIII transgene inducing formation of Tregs in one patient and effector T cells in another. Thus, though a xenogeneic transgene is utilized, these preclinical models actually provide the opportunity to elucidate how MHC differences between patients may influence their propensity to respond to transgene fVIII, especially in cases wherein CRM is detected. It should also be noted that exposure to any form of fVIII in severe hemophilia A patients that lack detectable CRM possess the capacity to elicit a humoral immune response. Similarly, for mild to moderate hemophilia A patients that demonstrate CRM, any parts of a therapeutic transgene fVIII that are not endogenously produced by the patient have the potential to induce an immune response.

Several preclinical studies also demonstrate that strain, vector dosing, transgene design, and promoter/enhancer elements can equally influence the immunological outcome to AAV-fVIII gene therapy. Similar to disparities in immune responsiveness to syngeneic transgene canine fVIII observed in the Queens and UNC colonies, hemophilia A mice on a BALB/c background are more tolerogenic to xenogeneic transgene human fVIII than those on a S129-C57BL/6 background. Moreover, dosing and promoter/enhancer element utilization has been shown to directly impact the overall immunological outcome to transgene fVIII. Particularly, infusion of certain doses of AAV-HCB-fVIII into S129-C57BL/6 mice that are prone to developing inhibitors to xenogeneic and syngeneic recombinant fVIII products failed to develop inhibitors to BDD human fVIII, ET3 and An53, while other synthetic promoters rendered these mice responsive to codon-optimized human fVIII. Nevertheless, the use of xenogeneic transgenes certainly adds a layer of complexity that may confound interpretation of immune responses to fVIII following AAV-fVIII gene therapy in preclinical models of hemophilia A.

### Immunobiology of the Hematopoietic System as a Gene Therapy Target

Hematopoietic stem cells (HSCs) are a rare population of multipotent precursors that possess the ability to self-renew and differentiate into a variety of cell lineages. As a result, HSCs provide the unique opportunity to create a continuous reservoir of transgene-expressing cells, and thereby steady expression of a therapeutic gene product. Moreover, as HSCs can differentiate into myeloid and lymphoid derived immune constituents, HSCs allow for the potential to induce life-long immune tolerance to transgene products. Successful immune tolerance induction following HSC directed gene therapy has been documented to occur for solid organ transplantation, allergy, autoimmunity, and a variety of other disease models with genetic abnormalities (i.e., hemophilia A and B) (22, 128–134).

Using various mouse models, it has been shown that one of the main mechanisms by which HSC directed gene therapy may mediate immune tolerance to transgene products is through central tolerance, a process that eliminates developing autoreactive lymphocytes (i.e., T cells and B cells) and promotes the generation of nTregs. To remove T cells that have high affinity for “self” antigens and facilitate the development of nTregs, peripheral tissue-specific antigens under the control of a transcriptional regulator (autoimmune regulator; AIRE) are presented on MHC molecules by medullary thymic epithelial cells (mTECs) to developing thymocytes (135, 136); mutations in the gene encoding AIRE result in autoimmune polyendocrinopathy-candidiasis-ectodermal dystrophy (APECED), a multiorgan autoimmune disorder caused by the release of “self” reactive T cells into the periphery (137). However, AIRE does not account for all peripheral “self” antigens, with reports indicating that AIRE induces expression of up to 1835 gene products in the thymus (138). As a result, peripheral dendritic cells migrate to the thymus and work in concert with mTECs to maximize removal of autoreactive thymocytes and generation of nTregs (139, 140). There are three distinct populations of dendritic cells that are indicated to contribute to T cell central tolerance: resident dendritic cells (CD8α^+^ SIRPα^−^), migratory dendritic cells (CD8α^−^ CD11b^+^ SIRPα^+^), and plasmacytoid dendritic cells (CD11c^int^ CD45RA^int^). While resident dendritic cells present “self” antigens derived from the blood or cross-presented from mTECs, migratory and plasmacytoid dendritic cells present peripherally acquired “self” antigens on MHC molecules to developing thymocytes (141–144).

The fate of autoreactive thymocytes is hypothesized to be based on the “Goldilocks” model, wherein the TCR signaling strength defined by “functional avidity” (based on affinity and duration of interaction) dictates the outcome for developing thymocytes (145, 146). Thymocytes with low affinity for “self” antigens maturate and egress to the periphery as conventional naïve T cells. Conversely, thymocytes expressing TCRs with high affinity for “self” peptide-MHC complexes can undergo clonal deletion (programmed cell death) or receptor editing to develop a new TCR with lower affinity for “self” antigens, though anergy (a state of non-responsiveness) has also been described to occur. Although Foxp3^+^ thymocytes have been identified in the human thymus, little is known about how these cells develop in humans. Moreover, while the exact factors that determine whether thymocytes with affinity for “self” antigen will become nTregs is not well-defined, it is suggested that thymocytes with intermediate affinity for “self” antigen develop into nTregs.

During B cell development, immature B cells expressing autoreactive B cell receptors (BCRs) are similarly negatively selected. As BCRs recognize epitopes in their native three-dimensional structure, B cell central tolerance necessitates “self” antigen expression within the bone marrow. Developing immature B cells that do not recognize “self” antigen in the bone marrow further maturate and migrate into the periphery. However, BCR recognition of multivalent “self” antigens, resulting in extensive BCR cross-linking, undergo receptor editing, a process wherein the B cell is given a second opportunity to produce a non-autoreactive BCR. If a subsequent autoreactive BCR is generated, the developing B cell undergoes clonal deletion. Conversely, B cells that weakly engage “self” antigens become anergic.

Although effective at removing most autoreactive lymphocytes, central tolerance is incomplete. Thus, peripheral tolerance is crucial for maintenance of immune tolerance. Peripheral tolerance is the mechanism by which autoreactive lymphocytes in the periphery are rendered incapable of subsequently responding to cognate “self” antigen. Mechanisms by which peripheral tolerance regulate immunity include, but are not mutually exclusive to, the differentiation of naïve conventional T cells into iTregs, induction of anergy, and clonal deletion. As CD4^+^ T cells are essential mediators of both cytolytic and humoral immune responses to protein immunogens, modulating CD4^+^ T cell immunity is an effective approach to induce or maintain peripheral tolerance. In the absence of danger signals and under basal conditions, dendritic cells express “self” peptide-MHC II complexes to maintain peripheral tolerance (147). T cells that recognize “self” antigen in the absence of co-stimulatory signals and/or presence of co-inhibitory signals are rendered anergic. Conversely, conventional naïve T cells that recognize cognate “self” peptide-MHC Class II complexes in the presence of weak co-stimulatory signals and immunomodulatory cytokines differentiate into iTregs. Thus, in the event that HSC directed gene therapy permits transgene expression by dendritic cells or in the bone marrow microenvironment, HSC-directed gene therapy has the potential to promote lifelong central and peripheral immune tolerance to therapeutic fVIII, and thereby represents one of the most attractive and promising cellular targets for fVIII gene therapy.

### HSC-Directed Preclinical Gene Therapy Studies

Retroviral vectors represent a family of versatile and now advanced gene-transfer vehicles that possess the enabling property of stable integration into the target cell genome. Commonly utilized examples of parent viruses include Moloney murine leukemia virus (MoMLV) and human immunodeficiency virus (HIV). In their recombinant form, each has a relatively large packaging capacity easily accommodating the BDD fVIII transgene sequence. Additionally, these vectors are capable of transducing a wide range of cell types both *in vivo* and *ex vivo*. MoMLV-based gamma-retroviral vectors have been used clinically in the treatment of X-linked severe combined immunodeficiency (X-SCID) disease (148). In this setting, gene therapy successfully cured the disease in the majority of patients. However, in early clinical trials using first generation vector designs, severe adverse events occurred due to insertional mutagenesis. The exact nature of these leukemogenic events remains unclear but is speculated to have resulted from a combination of factors including the site of viral integration near protooncogenes, vector payload, and cell processing protocol. Despite these adverse events, the X-SCID gene therapy story should be considered a success due to the dramatic clinical improvement achieved in the majority of patients without any other clinical options for treatment and certain early mortality.

Following clinical confirmation of the previously theoretical concern of insertional mutagenesis, extensive research in the area of retroviral vector design led to advancement of HIV-1-based LV vectors. LVs are extensively modified versions of HIV-1 that have most of the viral genes and regulatory sequences removed. In general, expression cassettes contain two long terminal repeats (LTR), an internal promoter, and the therapeutic transgene. Furthermore, LVs can be pseudotyped with envelope proteins from other viruses or synthetic components that facilitate directed tropism toward a variety of cell types. The resulting recombinant vector particles do not contain the genetic material necessary to direct replication upon entry into a target cell, but do retain the ability to integrate their genetic material and facilitate design-directed control of a therapeutic transgene product. Furthermore, LV integration events can be identified using state of the art genomics technology, and the relative abundance of each integrant can be tracked in real time clinically. Importantly, no evidence of pathogenic insertional mutagenesis by a LV has been observed to date in more than 200 subjects treated with LV-modified HSPC or T cell products (149). Recombinant retroviral vectors now have been approved for several congenital disease indications including SCID caused by adenosine deaminase deficiency and β-thalassemia, as well as cancer indications involving chimeric antigen receptors.

HSPCs were among the initial cellular targets for retroviral gene transfer because of their accessibility and clinical experience and utility. HSPC transplantation protocols have been refined over the past half century and have become a reliable way to extract, manipulate, and re-administer cells with long-term engrafting and expansion potential. Two primary populations of cells exist in the bone marrow and blood compartment. One is of mesenchymal lineage, which has the potential to differentiate into bone, cartilage, and adipose cells. The other is hematopoietic in origin, which populates the blood compartment including myeloid, lymphoid, and erythroid lineages. Evans and Morgan reported the initial finding that hematopoietic cells could be genetically modified by retroviral vectors to express human fVIII, albeit at insufficient levels to be detected in plasma (37). Subsequently, other groups demonstrated *in vitro* that lymphoid cells inefficiently biosynthesize and secrete fVIII compared to other cell types, including those of myeloid lineage (150–152). *In vitro*, genetically modified bone marrow-derived mesenchymal stem/progenitor cells produce high levels of fVIII (38). Furthermore, they are thought to have long-term engraftment potential, and thus have been a target in many preclinical studies incorporating retroviral vectors. However, in these studies, transient *in vivo* expression was observed possibly due to transcriptional silencing and/or transplanted cell death.

The first preclinical HSPC gene therapy study to achieve sustained correction of fVIII activity to therapeutic levels in transplanted mice was conducted by Hawley and colleagues (39). Subsequently, Sakata and colleagues demonstrated genetic modification of CD34^+^ cells using a simian immunodeficiency virus-based vector and detectable, albeit low, plasma human fVIII levels following transplantation into NOD/SCID mice (153). Several key findings were made in these early studies. First, BDD-fVIII transgenes can be stably transferred by recombinant retroviral vectors. Second, sustained expression and accumulation in plasma of fVIII is achievable through *ex vivo* transduction and HSPC transplantation into conditioned recipients. However, these early studies also identified that inefficient expression/biosynthesis of BDD-hfVIII is a hurdle to clinical translation.

**Figure 3:**
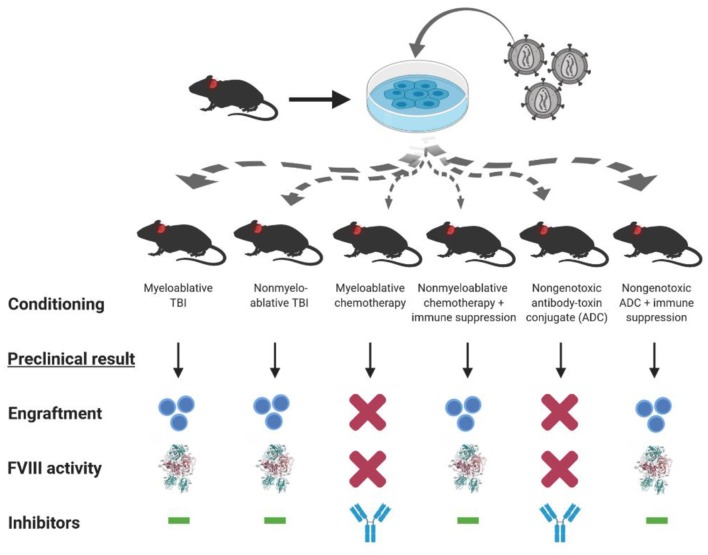
Conditioning dependent outcomes of preclinical HSPC LV-fVIII gene therapy. CD34^+^ HSPC isolated from hemophilia A or congenic mice are genetically modified *ex vivo* using LV-fVIII gene therapy. Transduced cells then are infused into naïve (or preimmunized with recombinant fVIII) hemophilia A mice in the presence or absence of various myeloablative and non-myeloablative conditioning regimens that are based on clinical transplantation protocols. Of the regimens tested in the preclinical stetting, myeloablative and non-myeloablative total body irradiation (TBI), or chemotherapy plus T cell immunosuppression (anti-thymocyte globulin or co-stimulation blockade), allowed for engraftment and corrective fVIII activity levels in the absence of inhibitor formation.

As mentioned previously, bioengineering fVIII for increased expression has become an increasingly active area of research by all key stakeholders. For example, early studies by our group demonstrated that BDD porcine (p)fVIII is expressed at levels 10–100-fold higher than BDD human fVIII from bone marrow-derived cell types transduced with retroviral vectors (16). Genetically modified murine HSPCs were shown to express high levels of BDD-pfVIII after transplantation into mice, and non-myeloablative conditioning was sufficient to facilitate engraftment of genetically modified HSPCs (16, 18). Subsequently, we demonstrated that non-myeloablative chemotherapy regimens incorporating immune suppression through T cell depletion or co-stimulation blockade also were successful at producing long-term engraftment, fVIII expression, and immune tolerance to endogenously produced or exogenously administered fVIII (Figure 3) (18, 94). Therefore, HSPC LV-fVIII gene therapy appears to be a promising approach with lifelong curative potential that can be accessible to all patients with hemophilia without age restriction, as both HSPC transplantation and HSPC gene therapy have been successfully utilized in children <1 year of age for other disease indications. Currently, the main limitation recognized for HSPC LV-fVIII gene therapy remains the toxicity-associated conditioning regimens that include transient immune suppression, risk of infection, and genotoxicity. Recently, our group and others have begun investigating non-genotoxic conditioning agents for utilization in HSPC transplantation and gene therapy [unpublished data and (154, 155)]. These agents take the form of antibody-drug conjugates (ADC) that possess immune and/or stem cell recognition and potent toxicity following cell internalization through the incorporation of toxins such as the ribosomal inactivating protein, saporin. Although the proof of concept data is impressive in terms of targeted stem cell depletion and facilitation of HSPC (both non-modified and genetically modified) engraftment, ongoing product development is needed to generate products suitable for clinical testing. However, it appears likely that ADC or similar technologies will revolutionize the safety and efficacy of HSPC transplantation and facilitate the implementation of HSPC LV gene therapy for a multitude of genetic diseases.

**Figure 4:**
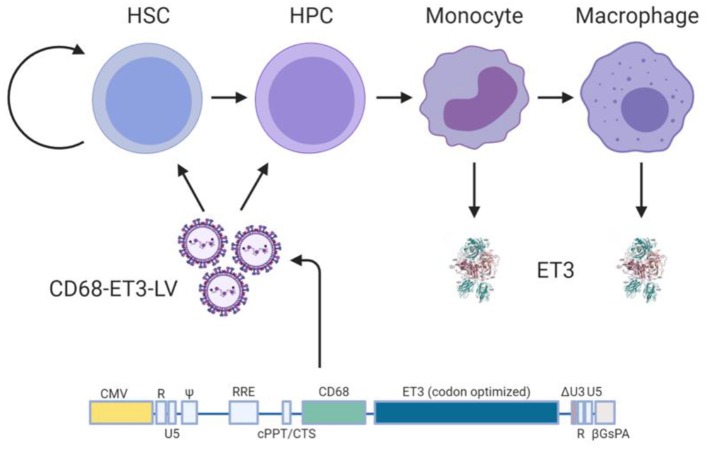
*Ex vivo* CD68-ET3-LV CD34^+^ clinical gene therapy paradigm. Autologous CD34^+^ HSPC are isolated from subjects with hemophilia A, genetically modified *ex vivo* using LV encompassing a codon optimized pfVIII transgene (ET3) under the monocyte lineage restricted promoter, CD68. Genetically modified HSPCs are then infused back into the subject following non-myeloablative conditioning with immune suppression. Post-administration of the genetically-modified autologous cell product, plasma fVIII levels, vector copy number in peripheral blood, and fVIII immunity status are followed.

In terms of HSPC LV-fVIII design and preclinical testing, we published a comprehensive set of preclinical studies supporting the clinical testing of an HSPC gene therapy for hemophilia A. The product candidate, referred to as CD68-ET3-LV CD34^+^, consists of autologous CD34^+^ cells transduced with a HIV-1-based, monocyte lineage-restricted, self-inactivating LV encoding the high-expression ET3 transgene (Figure 4) (19, 24). An Investigational New Drug (IND) application for this product candidate was recently cleared for clinical testing by the United States of America Food and Drug Administration. In the absence of validated preclinical immunogenicity models, directed immunogenicity testing was performed by comparative immunogenicity analysis of recombinant ET3 intravenously infused into E16 hemophilia A mice as well as *in silico* analysis of potential T cell epitopes. Overall, no significant differences were identified between ET3 and BDD human fVIII. To our knowledge, these studies represent the only specifically designed immunogenicity studies published for a bioengineered fVIII gene therapy candidate to date, despite the knowledge that all fVIII gene therapy products represent bioengineered versions of fVIII.

### Inhibitor Eradication via HSPC-Directed LV-fVIII Gene Therapy?

As mentioned previously, the theoretical clinical challenges posed by pre-existing fVIII immunity have precluded this subject population from participating in clinical gene therapy trials. Although AAV-fVIII clinical trials may soon open to the inhibitor population, little preclinical data support this approach and the existing data appear contradictory. Therefore, as an alternative approach to addressing this unmet clinical need, our group has studied HSPC LV-fVIII gene therapy in preclinical models with pre-existing immunity to human fVIII (17, 18, 22, 94). We discovered the need for potent immunosuppressive conditioning regimens to achieve stable, long-term engraftment in the pre-immunized setting as can be achieved with reduced intensity conditioning in naïve animals (Figure 3). Specifically, a requirement for either high-dose total body irradiation or chemotherapy plus immune suppression using anti-thymocyte globulin was necessary to facilitate engraftment and efficacy. We also demonstrated that inclusion of a high expression fVIII transgene not only restored curative plasma fVIII levels, but also permanently eradicated fVIII inhibitors. We continue to investigate novel conditioning agents (e.g., ADC) and immune-suppressing agents (e.g., T and B cell-blocking such as CTLA4-Ig) that should facilitate the application of HSPC LV-fVIII gene therapy to all persons with hemophilia A.

## Conclusions

Preclinical studies and early clinical data have yielded a wealth of evidence supporting the safety and efficacy of fVIII gene therapy. Moreover, these studies have provided essential information regarding factors that allow for successful fVIII gene therapy outcomes, including strategies for vector serotype, promoter/enhancer, dose, pre-transplantation conditioning regimens, and fVIII transgene optimization. As a result, fVIII gene therapy clinical trials have rapidly progressed over the past two decades. Recent fVIII gene therapy clinical trials demonstrate remarkable corrections in fVIII activity levels in the absence of inhibitor formation, and thereby provide optimism for a potential cure for hemophilia A. However, these clinical trials are restricted to adult PTPs without a history of inhibitors. As this subset of patients is inherently at low risk of developing inhibitors following fVIII exposure, current clinical trials do not provide indication for global usage of fVIII gene therapy. This is especially the case in previously untreated children and patients with pre-existing inhibitors, both of which may not benefit from AAV-based gene therapy, but could from HSPC LV-fVIII strategies. As highlighted in this review, limited preclinical data exist addressing the immunogenicity risk of fVIII gene therapy *a priori* and post-inhibitor development, though current studies provide strong evidence for the potential for gene therapy to mediate tolerance through formation of a Treg response. Thus, further examination of the mechanism(s) by which fVIII gene therapy can shift the balance from immunogenicity to tolerance will be critical to assess the immunological risk and/or benefit of gene therapy for PUPs and patients with pre-existing inhibitors. In addition, these studies likely will provide fundamental insight into how fVIII gene therapy can be manipulated to be utilized as an alternative to standard ITI. Finally, preclinical studies examining the longevity of fVIII gene therapy and gene therapy-mediated immune tolerance induction in PUPs as well as patients with pre-existing inhibitors will necessitate exploration, as both humoral and cellular immunity to vectors, especially AAV vectors, can preclude re-administration of fVIII gene therapy. However, since HSPC LV-fVIII approaches target stem cells, it is predicted that this approach can produce lifelong fVIII production. Therefore, gene therapy does offer the first potential and promising cure for hemophilia A. Moreover, as gene therapy consists of a single treatment event and even small increases in circulating fVIII plasma levels (>10 pM) can provide significant clinical benefits for patients with hemophilia A, gene therapy may be a more cost-effective option than factor replacement therapy for a large, worldwide population of patients with hemophilia A with limited access to treatment. Thus, as hemophilia A occurs in 1 in 4,000 male births, gene therapy possesses the capacity to revolutionize treatment for ~500,000 patients with hemophilia A worldwide.

## Author Contributions

SP, TL, HS, and CD drafted and edited the manuscript.

### Conflict of Interest

CD and HS are co-founders of Expression Therapeutics and own equity in the company. Expression Therapeutics owns the intellectual property associated with ET3. The terms of this arrangement have been reviewed and approved by Emory University in accordance with its conflict of interest policies. The remaining authors declare that the research was conducted in the absence of any commercial or financial relationships that could be construed as a potential conflict of interest.
